# Parametric Neural Network-Based Model Free Adaptive Tracking Control Method and Its Application to AFS/DYC System

**DOI:** 10.1155/2022/4579263

**Published:** 2022-01-06

**Authors:** Zhijun Fu, Yan Lu, Fang Zhou, Yaohua Guo, Pengyan Guo, Heyang Feng

**Affiliations:** ^1^Henan Key Laboratory of Intelligent Manufacturing of Mechanical Equipment, Zhengzhou University of Light Industry, Zhengzhou 450002, China; ^2^Research Center of Yutong Bus Co., Ltd., No. 66, Yuxing Road, Zhengzhou 450061, China; ^3^Department of Mechanical Engineering, North China University of Water Resources and Electric Power, No. 36, Beihuan Road, Zhengzhou 450045, China

## Abstract

This paper deals with adaptive nonlinear identification and trajectory tracking problem for model free nonlinear systems via parametric neural network (PNN). Firstly, a more effective PNN identifier is developed to obtain the unknown system dynamics, where a parameter error driven updating law is synthesized to ensure good identification performance in terms of accuracy and rapidity. Then, an adaptive tracking controller consisting of a feedback control term to compensate the identified nonlinearity and a sliding model control term to deal with the modeling error is established. The Lyapunov approach is synthesized to ensure the convergence characteristics of the overall closed-loop system composed of the PNN identifier and the adaptive tracking controller. Simulation results for an AFS/DYC system are presented to confirm the validity of the proposed approach.

## 1. Introduction

Nonlinearity and model uncertainty for practical nonlinear systems present great challenge for the controller design. Active chassis control system is a good instance of such kind of system. Integrated AFS/DYC system has become a very active field in advanced active chassis control system design as summarized in [1, 2]. The main control objective of the AFS/DYC system is to track the desired yaw rate and sideslip angle with the aim of achieving the satisfactory stability performance under different driving maneuvers. However, vehicle chassis system is an uncertain system in nature and incorporated unknown dynamics and disturbances, which bring great challenge for the controller design.

The identification of unknown nonlinear dynamic systems is often a prerequisite for successful controller design. Neural network, owing to their good generalization and nonlinear approximation ability, is widely used to identify model free nonlinear systems and exhibit higher performance compared to other identification methods. The reported neural network identifiers may be classified into two categories on the basis of the neural network structure used, namely, static neural network [3] and dynamic neural network [4, 5]. The main drawback of the static neural network is that the function approximation treatment makes it easy to fall into local optimum. Dynamic neural network method combines feedback information to provide an effective means to solve a wide range of identification problems. However, the structure of dynamic neural network lacks a unified form. The Hopfield network is a typical dynamic neural network in which every processing unit is connected to all other units [6]. A large number of neural network structures have been developed from Hopfield neural network, falling into two main categories, namely, high order neural networks [7] and multilayer dynamic neural network [8, 9]. Multiple nonlinear function in high order neural networks is used to approximate nonlinear dynamics, which brings the curse of dimensionality problem with the increase of order. Multilayer dynamic neural networks which contain additional hidden layers combined with a dynamic operator are not easy to design the online updating law. In general, popular learning rule such as backpropagation algorithm is used to design the online weight updating laws of dynamic neural networks, and then suitable candidate of Lyapunov function is proposed to ensure the stability of the system [10, 11]. In order to solve the locally minimal convergence problem caused by backpropagation algorithms, a novel updating law of multilayer dynamic neural networks is proposed in [12], where the global asymptotic error stability is guaranteed by defining a Lyapunov function candidate based on quadratic functions of the weights and the estimation errors. In [13], a model-based semi-Markov neural network is proposed. In [14, 15], the reported neural adaptive control designs are limited to a class of strict-feedback systems.

To get rid of model-based tracking controller design [16, 17], indirect adaptive control scheme is a widely used control strategy for model free nonlinear system [18, 19], which is achieved by a neural identifier or neuro observer to estimate the unknown system dynamics and an adaptive control law to minimize the tracking error. However, two issues are still needed to mention in this paper. Firstly, the state identification error is usually used to design the learning law for the existed neural identifier [20–22], which may affect the accuracy and convergence speed of the entire control loop owing to the inherent parameter drift problem. Secondly, most of the existed indirect adaptive control methods [23, 24] rely on the well-known linear separation principal to design the identifier and controller separately, which may affect the closed-loop stability when confronting the uncertain system dynamics. In this paper, we propose a new PNN-based indirect adaptive tracking control method for model free nonlinear systems. The notable contributions of the study are listed as follows:

1. A PNN identifier with a more parsimonious form is derived by extracting the parameter matrix of correlation weights multiplied by the correlation input and output state. Unlike the commonly used backpropagation learning law for neural network-based identification method, a novel parameter error-driven updating law is synthesized to ensure improved performance in terms of steady-state error.
2. Based on the identifier, we design the adaptive tracking control policy in terms with two terms, i.e., a feedback control term to compensate the identified nonlinearity and a sliding model control term to deal with the modeling error. The asymptotic convergence stability of the closed-loop system is proved by properly designing a composite Lyapunov function candidate.
3. Online adaptation property of the proposed adaptive tracking control method makes it very convenient for operating in practical application. The simulation results of an AFS/DYC system demonstrate the improved performance of the proposed method than the conventional neural network-based adaptive tracking control method.

The remainder of this paper is organized as follows. In Section 2, the PNN-based identification algorithm is given. The indirective adaptive tracking control policy is introduced in Section 3. Simulation results of an AFS/DYC system based on the nonlinear vehicle model are analyzed in Section 4. Finally, the conclusions are drawn in Section 5.

## 2. Identification Algorithm

Considering the following nonlinear systems:

(1) $$\begin{array}{c} \dot{x}=f\left(x,u\right), \end{array}$$

where *x* ∈ *R*^*n*^ is the state variable, *u* ∈ *R*^*p*^ is the input vector, and *f*(·) is the unknown continuous nonlinear smooth function.

It is well known that dynamic neural network can approximate the general nonlinear system (1) to any degree with the following form [25]:

(2) $$\begin{array}{c} {\dot{x}}_{i}=-a_{i}x_{i}+\sum_{j}w_{ij}S_{j}\left(x_{j},u_{j}\right), \end{array}$$

where *x*_*i*_ is the state of the *i*th neuron, *a*_*i*_ is the constant which is usually assumed to be known in advance, *w*_*ij*_ is the synaptic weight connecting the *j*th input to the *i*th neuron, and the nonlinear mapping *S*_*j*_ constitutes the *j*th state *x*_*i*_ and input *u*_*j*_ to the relational neuron.

**Figure 1:**
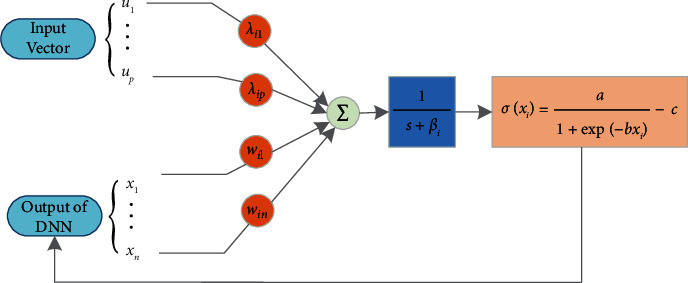
Block structure of the parametric neural network.

A more efficient PNN model with the simplest architecture has been introduced, such that

(3) $$\begin{array}{c} {\dot{x}}_{i}=-a_{i}x_{i}+\sum_{j=1}^{n}w_{ij}\sigma \left(x_{j}\right)+\sum_{j=1}^{p}\lambda_{ij}u_{j}, \end{array}$$

where *w*_*ij*_ and *λ*_*ij*_ are updated weights and *σ*(·) is the sigmoid function which is defined as *σ*(·)=*a*/(1+*e*^−*bx*^) − *c*, where *a, b,* and *c* are designed constants.  Figure 1 shows the block diagram of the PNN model (3).

**Remark 1 .**

The use of input affine neural network architecture (3) to approximate the nonautonomous systems (1) is advantageous, since many important nonlinear control schemes require input affine nonlinear models.

The PNN is formed by a single layer of *n* units as in equation (3). For the convenience of analysis, the vectorized expression of (3) is obtained with the following form:

(4) $$\begin{array}{c} \dot{x}=-ax+w\sigma \left(x\right)+\lambda u+\xi , \end{array}$$

where *x* ∈ *R*^*n*^ is the state vector, *a* ∈ *R*^*n*×*n*^ is the unknown matrix for the linear part of PNN model, *w* ∈ *R*^*n*×*n*^, *σ*(*x*)=[*σ*(*x*_1_),…,*σ*(*x*_*n*_)]^*T*^ ∈ *R*^*n*^, *λ* ∈ *R*^*n*×*m*^, *u* ∈ *R*^*p*^ is the input vector, and *ξ* denotes modeling error and disturbances and is assumed to be bounded $\left\|\xi \right\|\leq \bar{\xi }$.

Furthermore, we define the vector notations composed of unknown parameters of parametric dynamic neural network as *θ*=[*a*, *w*, *λ*]^*T*^ and the regressor vector as *ψ*=[*x*, *σ*(*x*), *u*]^*T*^, then the compact form of (4) becomes

(5) $$\begin{array}{c} \dot{x}=\theta^{T}\psi . \end{array}$$

**Remark 2 .**

Several adaptive identifiers have been proposed for system (6), where the adaptive laws are all designed by minimizing the residual identifier error (i.e., error between system state *x* and the identifier output $\hat{x}$) based on least square method or gradient method. However, the identifier weight convergence was not guaranteed. As indicated in [26], the convergence of the identifier weights is essential for the convergence of the control. This paper will present a novel adaptive law to directly identify the unknown parameters of PNN with compact form in (5).

Next, we will design an improved weight updating law to ensure the convergence of state identification error and parameters error. Thus, we define the filtered variables *x*_*f*_ and *ψ*_*f*_ of *x* and *ψ* as

(6) $$\begin{array}{c} \left\{\begin{array}{c} l{\dot{x}}_{f}+x_{f}=x,x_{f}\left(0\right)=0,\\ l{\dot{\psi }}_{f}+\psi_{f}=\psi ,\psi_{f}\left(0\right)=0, \end{array}\right. \end{array}$$

where *l* is the designed filter constant.

Then, from (5) and (6), we can get

(7) $$\begin{array}{c} x_{f}=\frac{x-x_{f}}{l}=\theta^{T}\psi_{f}. \end{array}$$

Further, we define the filtered regression matrix *E*(*t*) and *F*(*t*) vector as

(8) $$\begin{array}{c} \left\{\begin{array}{c} {\dot{E}}_{1}\left(t\right)=-\eta E_{1}\left(t\right)+\psi_{f}\left(t\right)\psi_{f}{\left(t\right)}^{T},\;E_{1}\left(0\right)=0,\\ {\dot{F}}_{1}\left(t\right)=-\eta F_{1}\left(t\right)+F_{f}^{T}\left(t\right){\left[\frac{\left(x\left(t\right)-x_{f}\left(t\right)\right)}{l}\right]}^{T},\;F_{1}\left(0\right)=0, \end{array}\right. \end{array}$$

where *η* is the designed filter constant.

From (8), one can get

(9) $$\begin{array}{c} \left\{\begin{array}{c} E_{1}\left(t\right)=\int e^{-\eta \left(t-r\right)}\psi_{f}\left(r\right)\psi_{f}^{T}\left(r\right)\text{d}r,\\ F_{1}\left(t\right)=\int e^{-\eta \left(t-r\right)}\psi_{f}\left(r\right){\left[\frac{\left(x\left(r\right)-x_{f}\left(r\right)\right)}{l}\right]}^{T}\text{d}r. \end{array}\right. \end{array}$$

**Definition 1 .**

(see [26]). A vector or matrix function Φ is persistently excited (PE) if there exist *τ* > 0, *ε* > 0, such that ∫_*τ*_^*t+τ*^Φ(*r*)Φ(*r*)^*T*^d*r* > *εI*,  ∀ *t* ≥ 0. Since Φ(*r*)Φ(*r*)^*T*^ is always positive semidefinite, the PE condition requires that its integral over any interval of time of length is a positive definite matrix.

**Remark 3 .**

If the repressor vector Φ is PE, then Φ_*f*_ defined in (6) is PE, because Φ_*f*_ is the filtered version of Φ in terms of a minimum strictly proper transfer function 1/(*ks*+1) in (6) as proved in [26]. Moreover, based on Definition 1, if Φ_*f*_ is PE, the inequality ∫_*τ*_^*t*+*τ*^Φ_*f*_^*T*^(*r*)Φ_*f*_(*r*)d*r* > *εI* is true for all *t* > 0, *ε* > 0. Then, ∫_*τ*_^*t*+*τ*^*e*^−*l*(*t* − *r*)^Φ_*f*_^*T*^(*r*)Φ_*f*_(*r*)d*r* > *εI* holds for all *t* > 0, *ε* > 0.

Considering the following identifier:

(10) $$\begin{array}{c} \dot{\hat{x}}={\hat{\theta }}^{T}\psi +Ke, \end{array}$$

where $e=x-\hat{x}$, $\hat{\theta }={\left[\hat{a},\hat{w},\hat{\lambda }\right]}^{T}$ and *K* > 0 is a designed parameter.

From (5) and (11), we can get

(11) $$\begin{array}{c} \dot{e}=\dot{x}-\dot{\hat{x}}=\theta^{T}\psi -{\hat{\theta }}^{T}\psi -Ke+\xi =-Ke+{\tilde{\theta }}^{T}\psi +\xi , \end{array}$$

where $\tilde{\Theta }=\Theta -\hat{\Theta }$ is the parameter identification error.

Finally, we denote another auxiliary vector as

(12) $$\begin{array}{c} M\left(t\right)=E\left(t\right)\hat{\Theta }-F\left(t\right), \end{array}$$

where $\hat{\Theta }$ is theta. It is clear that *M*(*t*) can be calculated based on equation (9).

**Remark 4 .**

From (8)–(10), we have $M\left(t\right)=E\left(t\right)\hat{\Theta }-F\left(t\right)=E\left(t\right)\hat{\Theta }-E\left(t\right)\Theta =-E\left(t\right)\tilde{\Theta }$, as can be seen that *M*(*t*) is composed of weights error $\tilde{\Theta }$, which is used to design the improved updating law in the next analysis.

Then, by using the auxiliary vector *M*(*t*), one can have the following improved updating law:

(13) $$\begin{array}{c} \dot{\hat{\theta }}=\Gamma \left[\psi e-\rho M\right], \end{array}$$

where Γ=Γ^*T*^ > 0 and *ρ* > 0 is positive constant.

**Theorem 1 .**

Consider system (1) with the identifier (11) and parameters adaptive law (13), then the convergent properties of identification error as well as parameters error can be obtained as follows:

- With the assumption that *ξ*=0, we have *e*, *θ* ∈ *L*_*∞*_ and lim_*t*⟶*∞*_*e*=0
- With the assumption that *ξ* is bounded, then we have *e*, *θ* ∈ *L*_*∞*_

**Proof**

Choose a Lyapunov function as

(14) $$\begin{array}{c} L_{I}=e^{T}Pe+\frac{1}{\Gamma }tr\left\{{\tilde{\theta }}^{T}P\tilde{\theta }\right\}. \end{array}$$

**Case 1 i.**

If *ξ*=0, then from (11)–(13) and $\dot{\tilde{\theta }}=-\dot{\hat{\theta }}$, one can get the differential of (14) as

(15) $$\begin{array}{c} {\dot{L}}_{I}=\left({\dot{e}}^{T}Pe+e^{T}P\dot{e}\right)+\frac{2}{\Gamma }tr\left\{{\dot{\tilde{\theta }}}^{T}P\tilde{\theta }\right\}\\ ={\left[{\tilde{\theta }}^{T}\psi -Ke\right]}^{T}Pe+e^{T}P\left({\tilde{\theta }}^{T}\psi -Ke\right)+\frac{2}{\Gamma }tr\left\{{\dot{\tilde{\theta }}}^{T}P\tilde{\theta }\right\}\\ =2e^{T}P{\tilde{\theta }}^{T}\psi -2e^{T}PKe-2\left(\psi e-\rho M\right)P\tilde{\theta }\\ =-2e^{T}PKe+2P{\tilde{\theta }}^{T}PM\\ =-2e^{T}PKe+2\rho {\tilde{\theta }}^{T}P\left(E\left(t\right)\hat{\Theta }-F\left(t\right)\right)\\ =-2e^{T}PKe+2\rho {\tilde{\theta }}^{T}P\left(E\left(t\right)\hat{\Theta }-E\left(t\right)\Theta \right)\\ =-2e^{T}PKe-2\rho {\tilde{\theta }}^{T}PE\left(t\right)\tilde{\theta }\leq 0. \end{array}$$

From (15), we know that *e*, *θ* ∈ *L*_*∞*_. Furthermore, one can infer from (11) that $\dot{e}\in L_{\infty }$. Based on the nonincreasing property of the function *V*, the integral of *V* on both sides from 0 to ∞ can be obtained:

(16) $$\begin{array}{c} \int_{0}^{\infty }\left(-2e^{T}PKe-2\rho {\tilde{\theta }}^{T}PE\left(t\right)\tilde{\theta }\right)=\left[V_{x}\left(0\right)-V_{x}\left(\infty \right)\right]<\infty . \end{array}$$

Therefore, *e* ∈ *L*_2_ can be obtained from (16). It can be concluded that *e* ∈ *L*_2_∩*L*_*∞*_ and $\dot{\Delta }x,\dot{\Delta }y\in L_{\infty }$. It is thus obtained from Barbalat's lemma [27] that lim_*t*⟶*∞*_*e*=0.

**Case 2 ii.**

For bounded *ξ*, by designing the same Lyapunov function as formula (14), one obtains

(17) $$\begin{array}{c} {\dot{L}}_{I}=\left({\dot{e}}^{T}Pe+e^{T}P\dot{e}\right)+\frac{2}{\Gamma }tr\left\{{\dot{\tilde{\theta }}}^{T}P\tilde{\theta }\right\}\\ ={\left[{\tilde{\theta }}^{T}\psi -Ke+\xi \right]}^{T}Pe+e^{T}P\left({\tilde{\theta }}^{T}\psi -Ke+\xi \right)+\frac{2}{\Gamma }tr\left\{{\dot{\tilde{\theta }}}^{T}P\tilde{\theta }\right\}\\ =2e^{T}P{\tilde{\theta }}^{T}\psi -2e^{T}PKe-2\left(\psi e-\rho M\right)P\tilde{\theta }+2e^{T}P\xi \\ =-2e^{T}PKe+2\rho {\tilde{\theta }}^{T}PM+2e^{T}P\xi \\ =-2e^{T}PKe+2\rho {\tilde{\theta }}^{T}P\left(E\left(t\right)\hat{\Theta }-F\left(t\right)\right)+2e^{T}P\xi \\ =-2e^{T}PKe+2\rho {\tilde{\theta }}^{T}P\left(E\left(t\right)\hat{\Theta }-E\left(t\right)\Theta \right)+2e^{T}P\xi \\ =-2e^{T}PKe-2\rho {\tilde{\theta }}^{T}PE\left(t\right)\tilde{\theta }+2e^{T}P\xi \\ \leq -2e^{T}PKe-2\rho {\tilde{\theta }}^{T}PE\left(t\right)\tilde{\theta }+e^{T}P\Lambda_{2}Pe+\xi^{T}\Lambda_{2}^{-1}\xi \\ \leq -\mu_{1}\left(\left\|e\right\|\right)-\mu_{2}\left(\left\|\tilde{\theta }\right\|\right)+\mu_{3}\left(\left\|\xi \right\|\right), \end{array}$$

where *μ*_1_, *μ*_2_, *μ*_3_ are positive constants and Λ_1_, Λ_2_ are positive definite matrixes.

It can be seen from (17) that *L*_*I*_ is input-to-state stability (ISS) Lyapunov function, so by Theorem 1 in [27], we can get the stability of the system such that if the model errors *ξ* is bounded, then the updating law (3.8) can make the identification procedure stable, i.e., *e*, *θ* ∈ *L*_*∞*_.

## 3. Adaptive Tracking Control

It can be seen from Section 2 that the proposed PNN identifier as depicted in Theorem 1 can be used to approximate the model free nonlinear system in equation (1), such that

(18) $$\begin{array}{c} \dot{x}=-\alpha \hat{x}+w\sigma \left(\hat{x}\right)+\lambda u+\xi , \end{array}$$

where *ξ* represents the modeling error and disturbance.

Considering the following time-varying reference trajectory, in the form of,

(19) $$\begin{array}{c} {\dot{x}}_{r}=f\left(x_{r},t\right). \end{array}$$

The goal of the adaptive tracking control is to make the system state of equation (1) conform to the state of the reference model in equation (19).

Hence, the error of trajectory tracking is described as

(20) $$\begin{array}{c} e_{c}\left(t\right)=x-x_{r}. \end{array}$$

Then, from equations (18)–(20), the error dynamic equation is obtained as

(21) $$\begin{array}{c} {\dot{e}}_{c}=-\alpha \hat{x}+w\sigma \left(\hat{x}\right)+\lambda u+\xi -f. \end{array}$$

The adaptive tracking control *u* consists of a feedback control term *u*_*f*_ and a sliding model control term *u*_*s*_ can be expressed as

(22) $$\begin{array}{c} u=u_{1}+u_{2}, \end{array}$$

where *u*_1_ is used to compensate the identified nonlinearity and *u*_2_ is used to deal with the modeling error. We define *u*_1_ as follows:

(23) $$\begin{array}{c} u_{1}=\lambda^{-1}\left[ax_{r}-w\sigma \left(\hat{x}\right)+f\right]. \end{array}$$

The control action *u*_2_ is designed by using the sliding mode control theory, such that

(24) $$\begin{array}{c} u_{2}=\lambda^{-1}\left[-ae_{c}-K_{c}\mathrm{sgn}\left(e_{c}\right)\right], \end{array}$$

where *K*_*c*_ > 0 is a designed parameter.

**Theorem 2 .**

For model free nonlinear system (1), using the identifier (10) with updating laws (13) and control policy (22), then the stability property lim_*t*⟶*∞*_*e*_*c*_=0 holds.

**Proof**

By considering the PNN identifier in Section 2 and the adaptive tracking controller together as a whole process, then we can design the composite Lyapunov function candidate as

(25) $$\begin{array}{c} L=L_{I}+L_{c}. \end{array}$$

In Theorem 1, we already prove *L*_*I*_ ≤ 0 and the stability properties (1) and (2). Now let us consider the Lyapunov function candidate *L*_*c*_ for control purpose, such that

(26) $$\begin{array}{c} L_{c}=e_{c}^{T}e_{c}. \end{array}$$

Substituting (23) into (21), we have

(27) $$\begin{array}{c} {\dot{e}}_{c}=\alpha e_{c}+\lambda u_{2}+\xi . \end{array}$$

Using (24) and (27), we obtain the time derivative of (26) as follows:

(28) $$\begin{array}{c} {\dot{L}}_{c}=2e_{c}^{T}{\dot{e}}_{c}=2e_{c}^{T}\left(\alpha e_{c}+\lambda u_{2}+\xi \right)=2e_{c}^{T}\left(-K_{c}\mathrm{sgn}\left(e_{c}\right)+\xi \right)\leq -2\left(K_{c}-\left\|\xi \right\|\right)\left\|e_{c}\right\|. \end{array}$$

If we choose $K_{c}>\bar{\xi }$, then ${\dot{L}}_{c}<0$. Hence, we have the stability property lim_*t*⟶*∞*_*e*_*c*_=0 and $\dot{L}={\dot{L}}_{L}+{\dot{L}}_{c}\leq 0$.

**Figure 2:**
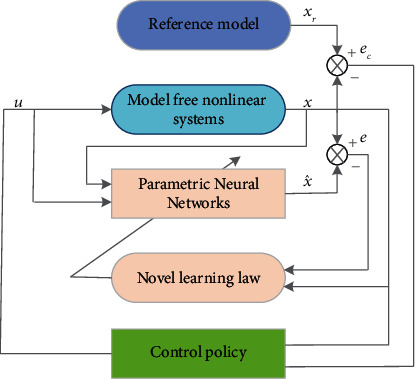
Adaptive tacking control scheme.

The overall structure of the PNN identifier and adaptive tracking controller is shown in Figure 2.

## 4. A Case Research: Application to an AFS/DYC Control System

**Figure 3:**
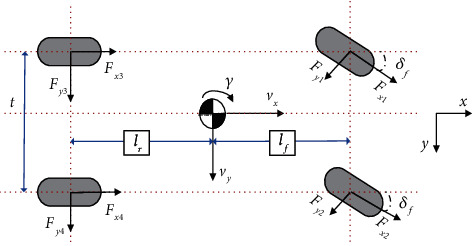
7-DOF nonlinear vehicle model.

A 7-DOF nonlinear vehicle model [28] (as shown in Figure 3) incorporates longitudinal and lateral tire forces calculated from Dugoff tire model which is used to verify the implementation of the proposed control algorithm. This model ignores heave, roll, and pitch motions but considers the lateral and longitudinal load transfers. The parameter notations mentioned above are described in Table 1.

Assume that the required yaw moment can be realized through the distribution of brake torques and steering angles of both front wheels are considered identical, then motion equations consisting of the external forces acting on the vehicle body in the longitudinal, lateral axes and the torques acting on the vertical axis can be written as

(29) $$\begin{array}{c} m{\dot{v}}_{x}=\left(F_{x1}+F_{x2}\right)\cos\left(\delta_{f}\right)+F_{x3}+F_{x4}-\left(F_{y1}+F_{y2}\right)\sin\left(\delta_{f}\right)+m\gamma v_{y},\\ m{\dot{v}}_{y}=F_{y3}+F_{y4}+\left(F_{x1}+F_{x2}\right)\sin\left(\delta_{f}\right)+\left(F_{y1}+F_{y2}\right)\cos\left(\delta_{f}\right)-m\gamma v_{x},\\ I_{Z}\dot{\gamma }=l_{f}\left(F_{x1}+F_{x2}\right)\sin\left(\delta_{f}\right)+l_{f}\left(F_{x1}+F_{x2}\right)\cos\left(\delta_{f}\right)-l_{r}\left(F_{y1}+F_{y2}\right)\\ +\frac{t}{2l_{f}\left(F_{x2}-F_{x1}\right)\cos\left(\delta_{f}\right)}+\frac{t}{2\left(F_{x4}-F_{x3}\right)}+\frac{t}{2\left(F_{y2}-F_{y1}\right)}. \end{array}$$

The tire force components (*F*_*xi*_ and *F*_*yi*_) in *x* and *y* directions can be calculated from the following transformation:

(30) $$\begin{array}{c} \left[\begin{array}{c} F_{xi}\\ F_{yi} \end{array}\right]=\left[\begin{array}{cc} \cos\delta_{i} & -\sin\delta_{i}\\ \sin\delta_{i} & \cos\delta_{i} \end{array}\right]\left[\begin{array}{c} F_{xwi}\\ F_{ywi} \end{array}\right],\;\left(i=1,2,3,4\right), \end{array}$$

where *F*_*xwi*_ and *F*_*ywi*_ are tire longitudinal and lateral forces in tire coordinate system, which are calculated from Dugoff tire model as follows. Here, a front steering vehicle is considered, i.e., *δ*_1_=*δ*_2_=*δ*_*f*_, *δ*_3_=*δ*_4_=0.

The normal load for each wheel can be expressed as

(31) $$\begin{array}{c} F_{z1}=\frac{mgl_{r}}{2\left(l_{f}+l_{r}\right)}-\frac{ma_{x}h}{2\left(l_{f}+l_{r}\right)}-\frac{ma_{y}h}{2t},\\ F_{z2}=\frac{mgl_{r}}{2\left(l_{f}+l_{r}\right)}-\frac{ma_{x}h}{2\left(l_{f}+l_{r}\right)}+\frac{ma_{y}h}{2t},\\ F_{z3}=\frac{mgl_{r}}{2\left(l_{f}+l_{r}\right)}+\frac{ma_{x}h}{2\left(l_{f}+l_{r}\right)}-\frac{ma_{y}h}{2t},\\ F_{z4}=\frac{mgl_{r}}{2\left(l_{f}+l_{r}\right)}+\frac{ma_{x}h}{2\left(l_{f}+l_{r}\right)}+\frac{ma_{y}h}{2t}. \end{array}$$

Dugoff tire model is selected to calculate longitudinal tire forces and lateral tire forces because it requires fewer coefficients and is relatively simple compared to the Magic Formula model. Moreover, it allows the use of independent values for tire cornering stiffness and longitudinal stiffness. Dugoff tire model can be defined as

(32) $$\begin{array}{c} F_{xwi}=C_{xi}\frac{\sigma_{i}}{1+\sigma_{i}}f\left(\lambda \right),\\ F_{ywi}=C_{yi}\frac{\tan\;\alpha_{i}}{1+\sigma_{i}}f\left(\lambda \right), \end{array}$$

where

(33) $$\begin{array}{c} \lambda =\frac{\mu F_{zi}\left(1+\sigma_{i}\right)}{2{\left\{{\left(C_{xi}\sigma_{i}\right)}^{2}+{\left(C_{yi}\tan\;\alpha_{i}\right)}^{2}\right\}}^{1/2}},\\ f\left(\lambda \right)=\left\{\begin{array}{cc} \left(2-\lambda \right)\lambda , & \left(\lambda <1\right),\\ 1, & \left(\lambda >1\right). \end{array}\right. \end{array}$$

The slip angle at each tire can be defined as

(34) $$\begin{array}{c} \alpha_{1}=\delta_{f}-\tan^{-1}\frac{v_{y}+l_{f}\gamma }{v_{x}-0.5t\gamma },\\ \alpha_{2}=\delta_{f}-\tan^{-1}\frac{v_{y}+l_{f}\gamma }{v_{x}+0.5t\gamma },\\ \alpha_{3}=-\tan^{-1}\frac{v_{y}-l_{f}\gamma }{v_{x}-0.5t\gamma },\\ \alpha_{4}=-\tan^{-1}\frac{v_{y}-l_{f}\gamma }{v_{x}+0.5t\gamma }. \end{array}$$

The wheel slip ratio at each tire can be described as

(35) $$\begin{array}{c} \sigma_{i}=\frac{R_{w}\omega_{wi}-v_{xi}}{\max\left(R_{w}\omega_{wi},V_{xi}\right)}. \end{array}$$

The wheel rotation dynamics can be given as

(36) $$\begin{array}{c} J_{wi}{\dot{\omega }}_{wi}=T_{dw\;i}-T_{bwi}-R_{w}F_{xi}. \end{array}$$

According to [28], the desired reference model is based on a 2-DOF single track vehicle model in steady-state condition and is usually expressed as

(37) $$\begin{array}{c} {\dot{x}}_{r}=A_{r}x_{r}+E_{r}\delta_{f}, \end{array}$$

where $x_{r}={\left[\begin{array}{cc} \beta_{r} & \gamma_{r} \end{array}\right]}^{T}$, *β*_*r*_ denotes the sideslip angle, *γ*_*r*_ denotes the yaw rate, $A_{r}=\left[\begin{array}{cc} -1/\tau_{\beta } & 0\\ 0 & -1/\tau_{\gamma } \end{array}\right]$, *τ*_*r*_ and *τ*_*β*_ are the designed time constants for yaw rate and sideslip angle, respectively, *δ*_*f*_ represents the steering input of the driver, $E_{r}={\left[\begin{array}{cc} \left(1-\left(ml_{f}/2\left(l_{f}+l_{r}\right)l_{r}C_{r}\right)v_{x}^{2}/1+\left(m/\left(l_{f}+l_{r}\right)\right)\left(\left(l_{f}/2C_{r}\right)-\left(l_{r}/2C_{f}\right)\right)v_{x}^{2}\right)\left(l_{r}/\left(l_{f}+l_{r}\right)\right) & \left(\left(v_{x}/l_{f}+l_{r}\right)/1+\left(m/\left(l_{f}+l_{r}\right)\right)\left(\left(l_{f}/2C_{r}\right)-\left(l_{r}/2C_{f}\right)\right)v_{x}^{2}\right) \end{array}\right]}^{T}$, and *C*_*r*_*C*_*f*_ are the cornering stiffness of the front and rear wheels.

**Figure 4:**
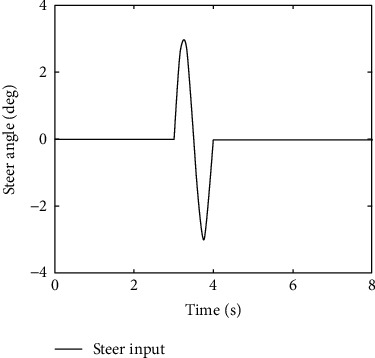
Steer input.

**Figure 5:**
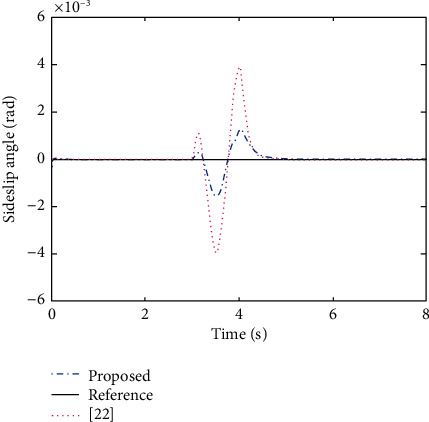
Control result of sideslip angle.

**Figure 6:**
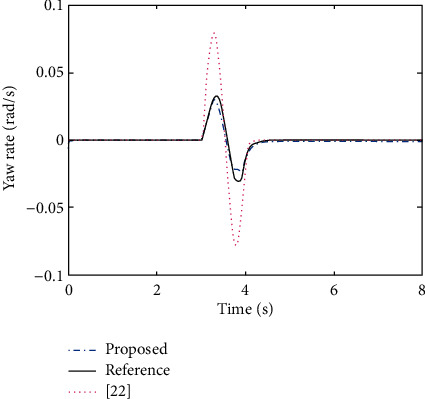
Control result of yaw rate.

The main objective of AFS/DYC control is to design a proper controller to keep the vehicle stable on the desired path, i.e., making the actual vehicle yaw rate and sideslip angle obtained from (29) to follow the desired responses obtained from (37). Here, the PNN identifier (10) with updating laws (13) and control policy (22) are selected as the AFS/DYC controller. In order to make a comparation with the commonly used AFS/DYC controller as showed in [28], we selected the same parameters as *m* = 1704 kg, *C*_*f*_ = 63224 N/rad, *C*_*r*_ = 84680 N/rad, *I*_*z*_ = 3048 kg·m^2^, *l*_*f*_ = 1.135 m, *l*_*r*_ = 1.555 m, and *μ*=0.8. In addition, the sine with dwell steer input, as shown in Figure 4, is used to verify the improved performance of the proposed method. It should be pointed out that the ideal sideslip angle for vehicle stability control should be selected as small as possible, and it is usually selected as zero. From Figures 5 and 6, one can easily find that the proposed adaptive tracking control method has better tracking performance with smaller tracking error and faster convergence rate to the steady state compared with the commonly used method as claimed in [28]. Therefore, we concluded that model free property of the proposed adaptive tracking control method provides a more effective solution for the integrated AFS/DYC controller design and can greatly enhance the vehicle handling and stability performances.

To show the identification performance of the proposed algorithm, the performance index root mean square (RMS) for the states error has been adopted for the purpose of comparison.

(38) $$\begin{array}{c} \text{RMS}=\sqrt{\sum_{i=1}^{n}\frac{e^{2}\left(i\right)}{n}}, \end{array}$$

where *n* is the number of the simulation steps and *e*(*i*) is the difference between the state variables in model and system at *i*^th^ step.

The RMS values of all state variables, as shown in Table 2, demonstrate that the identification performance has been improved compared to those in [28].

## 5. Conclusions

In this paper, a model free identification and adaptive tracking control method based on a parametric neural network (PNN) is proposed. The main contributions of the paper lie in the following aspects. First, the compact PNN form is derived by extracting the parameter matrix of correlation weight multiplied by the correlation input and output state, which simplifies the training problem and leads to more efficient models. Second, the filtered parameters error is introduced in the updating law, which can avoid the parameter drift problem and ensure the accuracy and rapidity of identification. Third, an adaptive tracking controller consists of a feedback control term to compensate the identified nonlinearity and a sliding model control term to deal with the modeling error is established. The stability of the overall closed-loop system is proved by designing a composite Lyapunov candidate. Finally, the application to AFS/DYC system is presented to verify the validity of the proposed methods.

## Figures and Tables

**Table 1:** Description of vehicle parameters.

Parameters	Description
*m*	Vehicle mass
*I*_z_	Yaw moment of inertia
*l*_*f*_, *l*_r_	Distance from CG to front axle and rear axle
*C*_*yi*_	Tire lateral stiffness
***C***_*xi*_	Tire longitudinal stiffness
*h*	CG height
*v*_*x*_, *v*_*y*_	Vehicle longitudinal and lateral speed
*a*_*x*_, *a*_*y*_	Vehicle longitudinal and lateral acceleration
*F*_*yf*_, *F*_*yr*_	Combined front and rear tire lateral force
*F*_*zi*_	Normal force of *i*th wheel
*g*	Gravity acceleration
*R*_*w*_, *J*_*w*_	Wheel rolling radius, moment of inertia
*ω*_*wi*_	Wheel angular speed
*T*_*bwi*_	Active brake torque
*T*_*dwi*_	Driving torque
*t*	Wheel track width
*μ*	Friction coefficient between tire and road
*γ*	Yaw rate about *z* axis
*α*_*i*_, *σ*_*i*_	The *i*th wheel slip angle, slip ratio
*δ*_*f*_	Front wheel steering angle

**Table 2:** The RMS values for tracking errors (10–4).

	Sideslip angle	Yaw rate
Proposed	2.885	3.963
Reference [28]	7.471	15.23

## Data Availability

Data supporting the results of this study can be provided as required.
